# Profiling Epidermal Growth Factor Receptor and Heregulin Receptor 3 Heteromerization Using Receptor Tyrosine Kinase Heteromer Investigation Technology

**DOI:** 10.1371/journal.pone.0064672

**Published:** 2013-05-20

**Authors:** Mohammed Akli Ayoub, Heng B. See, Ruth M. Seeber, Stephen P. Armstrong, Kevin D. G. Pfleger

**Affiliations:** 1 Laboratory for Molecular Endocrinology-GPCRs, Western Australian Institute for Medical Research (WAIMR) and Centre for Medical Research, The University of Western Australia, Nedlands, Western Australia, Australia; 2 Dimerix Bioscience Pty Ltd, Nedlands, Western Australia, Australia; 3 Protein Research Chair - Department of Biochemistry, College of Science, King Saud University, Riyadh, Kingdom of Saudi Arabia; Hungarian Academy of Sciences, Hungary

## Abstract

Heteromerization can play an important role in regulating the activation and/or signal transduction of most forms of receptors, including receptor tyrosine kinases (RTKs). The study of receptor heteromerization has evolved extensively with the emergence of resonance energy transfer based approaches such as bioluminescence resonance energy transfer (BRET). Here, we report an adaptation of our Receptor-Heteromer Investigation Technology (Receptor-HIT) that has recently been published as the G protein-coupled receptor (GPCR) Heteromer Identification Technology (GPCR-HIT). We now demonstrate the utility of this approach for investigating RTK heteromerization by examining the functional interaction between the epidermal growth factor (EGF) receptor (EGFR; also known as erbB1/HER1) and heregulin (HRG) receptor 3 (HER3; also known as erbB3) in live HEK293FT cells using recruitment of growth factor receptor-bound protein 2 (Grb2) to the activated receptors. We found that EGFR and HER3 heteromerize specifically as demonstrated by HRG inducing a BRET signal between EGFR/Rluc8 and Grb2/Venus only when HER3 was co-expressed. Similarly, EGF stimulation promoted a specific BRET signal between HER3/Rluc8 and Grb2/Venus only when EGFR was co-expressed. Both EGF and HRG effects on Grb2 interaction are dose-dependent, and specifically blocked by EGFR inhibitor AG-1478. Furthermore, truncation of HER3 to remove the putative Grb2 binding sites appears to abolish EGF-induced Grb2 recruitment to the EGFR-HER3 heteromer. Our results support the concept that EGFR interacts with Grb2 in both constitutive and EGF-dependent manners and this interaction is independent of HER3 co-expression. In contrast, HER3-Grb2 interaction requires the heteromerization between EGFR and HER3. These findings clearly indicate the importance of EGFR-HER3 heteromerization in HER3-mediated Grb2-dependent signaling pathways and supports the central role of HER3 in the diversity and regulation of HER family functioning.

## Introduction

Cell surface receptors promote and control vital physiological functions and constitute the major targets for drugs used to treat various diseases. Receptor tyrosine kinases (RTKs) are among the most extensively studied receptors due to their involvement in the control of cell proliferation, survival and differentiation. The type 1 RTK class is the HER/erbB receptor family and comprises four members, epidermal growth factor (EGFR, also known as erbB-1 or HER1, which is the most studied and characterized of the family), erbB-2/HER2, erbB-3/HER3, and erbB-4/HER4 [1]–[5]. RTKs are single chain transmembrane polypeptide proteins composed of three different domains: (i) the extracellular domain where the ligand binds the receptor, (ii) the transmembrane domain, and (iii) the cytoplasmic domain [1]–[5]. The cytoplasmic domain in turn consists of the juxtamembrane region, the tyrosine kinase domain that phosphorylates tyrosine residues, and the C-terminal region containing tyrosine residues that are themselves phosphorylated following ligand binding [4]. This autophosphorylation constitutes the key step linking RTK activation with multiple intracellular proteins containing Src homology 2 (SH2) domains, such as Chk, Grb2, Shc, and PI3-kinase. These adaptor proteins are then involved in a large protein interaction network that in turn activates various signal transduction molecules, including small G protein Ras, protein kinase B (PKB or Akt), the tyrosine kinase Src, mitogen- and stress-activated protein kinases, c-Jun kinase, and signal transducers and activators of transcription (STATs) [1]–[5].

The HER receptor family is of particular importance due to the link between abnormal expression and function of these receptors and many types of cancer [5]–[8]. Indeed, the dysregulation in erbB-mediated signaling has been shown to have major consequences on cell proliferation, apoptosis, angiogenesis, and migration. Moreover, the overexpression of erbB members has been observed in various human cancers [1], [3], [9]. Therefore, the investigation of RTK function is of considerable interest for drug discovery and cancer therapy programs based on the development of small molecule antagonists or antibodies blocking RTK-dependent signaling and responses.

Furthermore, one of the major characteristics of the HER receptor family is their heteromerization, which results in diverse HER-mediated cell signaling pathways [5], [7], [10], [11]. For instance, heteromerization is proposed to provide additional phosphotyrosine residues for the recruitment of various adaptor proteins and effectors inducing distinct patterns of receptor phosphorylation and downstream signaling [4], [5]. Traditionally with this family, ligand-induced dimerization has been considered to be the key step in mediating signaling following receptor activation, by positioning the two cytoplasmic domains of the receptors such that tyrosine transphosphorylation can occur. However, more recently it has been suggested that ligand binding results in conformational change in pre-existing complexes [12], [13]. To conciliate the different hypotheses, a systematic analysis of HER monomers versus dimers in various EGFR and HER2 expressing cell lines has shown that the degree of pre-formed and ligand-induced receptor dimerization depends on receptor expression levels and their distribution, which may affect the receptor-ligand binding properties [14]. There is also evidence for higher-order complex formation, with Clayton et al. suggesting that tetramers and higher-order oligomers of EGFR are the dominant activated species [15], building on their earlier work suggesting EGFR activation involves a dimer to tetramer transition [16].

The importance of RTK dimerization/oligomerization in physiology and pathophysiology is illustrated by various receptor complexes that can exist depending on the concentrations of the receptors expressed, the concentrations of particular ligands and some intrinsic degree of dimer selectivity [3], [17], [18]. Furthermore, it has been reported that HER heteromers are more potent in signal transduction than homomers. This is especially true when considering the heteromerization between HER2 and HER3, since HER2 enhances and stabilizes dimerization but has no ligand and HER3 can recruit novel proteins, but apparently lacks kinase activity [4], [8], [10], [17], [19]. Many studies have indicated that HER2, through its heteromerization with other HER members (essentially HER3), constitutes the key element in regulating and diversifying HER-mediated signaling as well as HER-linked cancer [20], [21]. As for HER3, it is known to differ from other HER members because of the absence of intrinsic catalytic kinase activity and a possible defect in forming homomers at the cell surface [22]–[24]. Therefore, HER3 is thought to be an activator for the HER family and recent studies reported that HER3 may also play a role in HER function and signaling through its heteromerization with other members including EGFR [11], [25]–[27].

**Figure 1:**
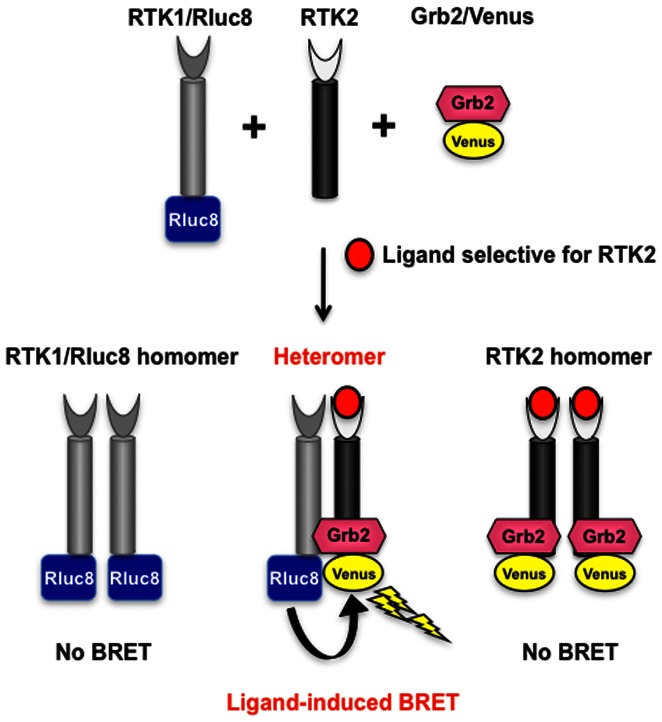
Receptor-HIT and its application to study RTK heteromerization (RTK-HIT) using BRET. Cells co-expressed RTK1 fused to a BRET donor (Rluc8), untagged RTK2, and a RTK signaling or adaptor protein tagged with a BRET acceptor (Venus), exemplified here by Grb2/Venus. After cell stimulation with the RTK2-selective agonist, only the recruitment of Grb2/Venus to the RTK1/Rluc8-RTK2 heteromer can be detected by BRET resulting from the physical proximity between RTK1/Rluc8 and Grb2/Venus.

We have adapted our Receptor-HIT technology, previously exemplified with GPCRs as ‘GPCR-HIT’ [28]–[33], to investigate the heteromerization between EGFR and HER3 in real-time and in live HEK293FT cells using the BRET platform to measure the ligand-induced recruitment of Grb2 to the activated receptor complex. One receptor was fused to the BRET donor, a variant of *Renilla* luciferase known as Rluc8 (shown as RTK1/Rluc8) and Grb2 was fused to the yellow fluorescent protein Venus as BRET acceptor (Grb2/Venus). Both were co-expressed with a second receptor (shown as RTK2) that was untagged with respect to BRET (Figure 1). The heteromerization between RTK1 and RTK2 was then assessed by measuring ligand-induced BRET between RTK1/Rluc8 and Grb2/Venus upon selective activation of RTK2, in a similar manner to that reported for GPCRs recruiting β-arrestin 2 [29], [31]–[33]. We compared these findings to those observed upon treating with a ligand selective for RTK1/Rluc8, in the presence and absence of RTK2, keeping in mind that such signals are likely to be largely due to RTK1/Rluc8 homomer activation in the case of EGFR.

## Materials and Methods

### Materials

EGFR, HER3 and Grb2 cDNAs were obtained from Origene (Rockville, MD, USA). EGFR and HER3 were subcloned into pcDNA3-Rluc8 prepared previously from cDNA kindly provided by Andreas Loening and Sanjiv Gambhir (Stanford University, CA, USA). Grb2 was subcloned into pcDNA3-Venus prepared previously from pcC2-Venus kindly provided by Atsushi Miyawaki (RIKEN Brain Science Institute, Wako-city, Japan). HER3trunc and HER3trunc/Rluc8 truncated after codon 1020 (corresponding to residue 1001 of the mature protein, thereby removing the C-terminal region but retaining the catalytically-inactive kinase domain [25]) were made by generating PCR fragments with and without a stop codon, from the internal BamHI site at nucleotide position 2183 of HER3 to position 3060. Via subcloning, these cDNA fragments were subsequently used to replace the C terminus of the full-length constructs (with or without Rluc8). Ligands used were EGF, Heregulin-β1 (HRG; Peprotech, Rocky Hill, NJ, USA) and AG-1478 hydrochloride (Tocris, Bristol, UK).

### Cell culture and transfection

HEK293FT cells (Life Technologies) were maintained at 37°C in 5% CO_2_ and complete media (Dulbecco's modified Eagle's medium (DMEM) containing 0.3 mg/ml glutamine, 100 IU/ml penicillin and 100 µg/ml streptomycin (GIBCO BRL, Carlsbad, CA, USA)) supplemented with 10% foetal calf serum (FCS) and 400 µg/ml Geneticin (Gibco). Transient transfections were carried out 24 h after seeding about 550,000 cells/well of a 6-well plate. Genejuice (Merck, Kilsyth, Australia) transfection reagent was used according to the manufacturer's instructions. Cells were harvested with 0.05% Trypsin-EDTA (Gibco).

### Ligand-induced BRET assays

**Figure 2:**
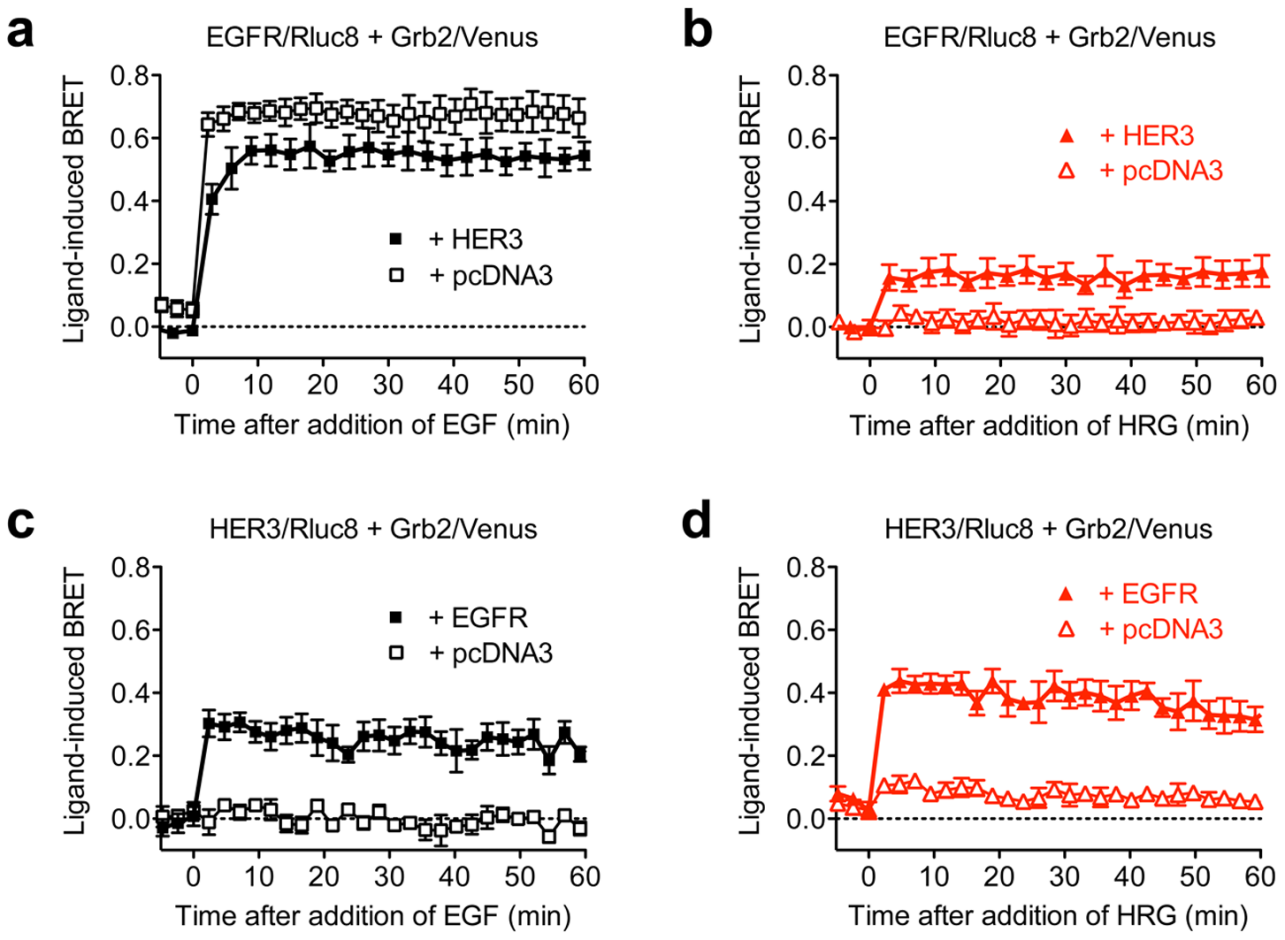
Kinetic analysis of EGF and HRG-induced recruitment of Grb2 to complexes containing EGFR and/or HER3. HEK293FT cells expressing EGFR/Rluc8 and Grb2/Venus, with and without HER3, were treated with 1 µM EGF (**a**) or HRG (**b**). Similarly, cells expressing HER3/Rluc8 and Grb2/Venus, with and without EGFR, were again treated with 1 µM EGF (**c**) or HRG (**d**). BRET was measured in real-time and live cells before and after stimulation with the agonists as indicated. Data represent mean ± SEM of 3–7 independent experiments.

HEK293FT cells were transiently transfected with cDNA encoding a RTK fused to Rluc8 (RTK/Rluc8) and Grb2 fused to Venus (Grb2/Venus), along with a second RTK that was untagged with respect to BRET signaling, or empty vector (pcDNA3). Following initial titration of cDNA amounts (data not shown), two ratios were selected for the heteromer combinations shown in Figure 2. For a 6-well plate, 0.1, 0.3 and 0.5 µg/well were used for the combination of EGFR/Rluc8, Grb2/Venus and HER3 and 0.1, 0.3 and 0.1 µg/well were used for the combination of HER3/Rluc8, Grb2/Venus and EGFR. 24 h post-transfection, cells were harvested into 96-well plates (Nunc) and 48 h post-transfection, cells were incubated at 37°C, 5% CO_2_ for 2 h with 30 µM EnduRen (Promega) to ensure substrate equilibrium was reached. BRET measurements were taken at 37°C using the VICTOR Light plate reader with Wallac 1420 software (PerkinElmer, Glen Waverley, Victoria, Australia). Filtered light emissions were sequentially measured at 400–475 and 520–540 nm. The BRET signal was calculated by subtracting the ratio of 520–540 nm emission over 400–475 nm emission for a vehicle-treated cell sample from the same ratio for a second aliquot of the same cells treated with agonist, as described previously [34], [35]. In this calculation, the vehicle-treated cell sample represents the background, eliminating the requirement for measuring a donor-only control [34], [35]. For these BRET kinetic assays, the final pretreatment reading is presented at the zero time point (time of ligand/vehicle addition). The situation where addition of ligand specific for the untagged RTK results in a ligand-induced BRET signal indicates Grb2 binding specifically to a heteromer complex [36].

### Data presentation and analysis

Data were analyzed using Prism 5 graphing software (GraphPad, La Jolla, CA, USA). Sigmoidal curves were fitted to the dose-response data using non-linear regression (log(inhibitor) versus response for the AG-1478 inhibition curves). Statistical analysis to compare pEC_50_ or pIC_50_ values was carried out using one-way ANOVA followed by Tukey's multiple comparison post-test.

## Results

### Kinetic analysis of the interaction between EGFR and HER3 using RTK-HIT

To investigate the functional interaction between EGFR and HER3 using RTK-HIT as shown in Figure 1, various combinations of Rluc8-tagged and untagged EGFR and HER3 were co-expressed with Venus-tagged Grb2 and real-time kinetic analysis carried out (Figure 2). We found that the co-expression of HER3 with EGFR/Rluc8 and Grb2/Venus did not substantially affect the rapid and very strong BRET increase promoted by EGF when compared to cells co-expressing only EGFR/Rluc8 and Grb2/Venus (Figure 2a). In contrast, HER3 co-expression was required for an HRG-induced BRET signal to be observed between EGFR/Rluc8 and Grb2/Venus (Figure 2b). In cells co-expressing HER3/Rluc8 and Grb2/Venus, no or little BRET signal was observed when cells were stimulated with either EGF (Figure 2c) or HRG (Figure 2d) respectively, consistent with minimal interaction between HER3 and Grb2. Interestingly, when EGFR was co-expressed with HER3/Rluc8 and Grb2/Venus, a strong BRET increase was observed upon stimulation with either EGF (Figure 2c) or HRG (Figure 2d). Taken together, these data indicate a functional interaction between EGFR and HER3 (heteromerization) enables EGF and HRG to promote Grb2 recruitment to the EGFR-HER3 heteromer. In fact, our data clearly demonstrate that the interaction between EGFR and HER3 is crucial for HER3 to functionally interact with Grb2.

### Dose-response analysis of the interaction between EGFR and HER3 using RTK-HIT

**Figure 3:**
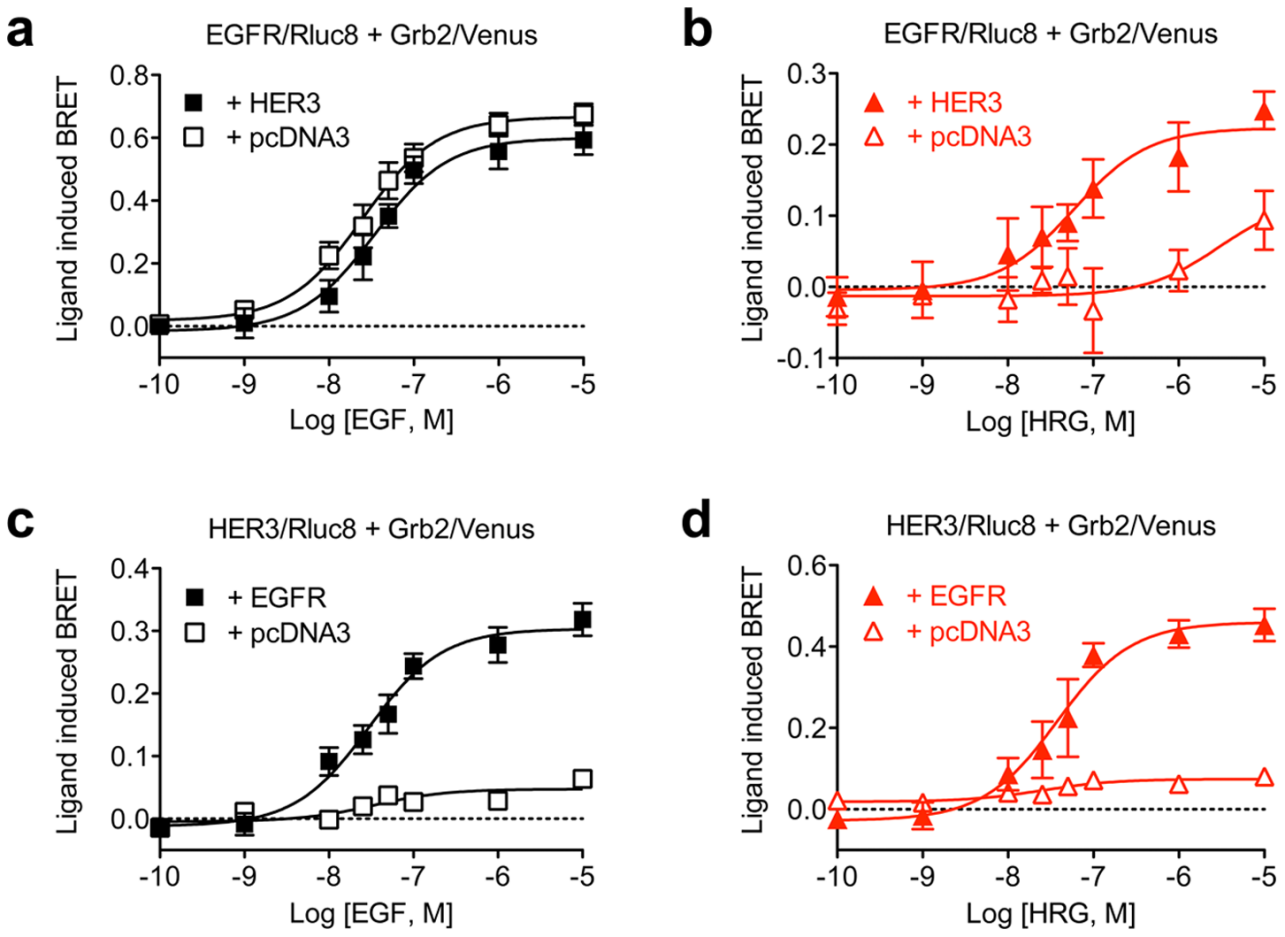
Dose-response analysis of EGF and HRG-induced recruitment of Grb2 to complexes containing EGFR and/or HER3. HEK293FT cells expressing EGFR/Rluc8 and Grb2/Venus, with and without HER3, were treated with increasing concentrations of EGF (**a**) or HRG (**b**). Similarly, cells expressing HER3/Rluc8 and Grb2/Venus, with and without EGFR, were again treated with increasing concentrations of EGF (**c**) or HRG (**d**). BRET was measured in live cells before and after stimulation with the agonists as indicated, with the data shown generated after about 25 minutes of agonist stimulation**.** Data represent mean ± SEM of 3–9 independent experiments.

To further profile the functional interaction between EGFR and HER3, we analyzed the dose-response effect of EGF and HRG on BRET signal using similar combinations to those described in Figure 2. The dose-response profile of EGF on BRET between EGFR/Rluc8 and Grb2/Venus was unchanged regardless of whether HER3 was co-expressed or not (Figure 3a; Table 1). However, the dose-dependent effect of HRG on BRET between EGFR/Rluc8 and Grb2/Venus was only observed when HER3 was co-expressed (Figure 3b) consistent with EGFR-HER3 interaction. As expected from the kinetic curves shown in Figure 2c and d, the increasing concentrations of both EGF (Figure 3c) and HRG (Figure 3d) nicely promoted increased BRET between HER3/Rluc8 and Grb2/Venus only when EGFR was co-expressed, with similar potencies between EGF and HRG (Table 1). These dose-response data indicate that the functional interaction between EGFR and HER3 is crucial for HRG to promote Grb2 recruitment via HER3 activation. In addition, the data clearly demonstrate that the recruitment of Grb2 to the EGFR-HER3 heteromer exclusively depends on the activation of at least one receptor within the heteromer.

**Table 1:** pEC_50_ values for EGF and HRG.

*Transfections*	*EGF*	*HRG*
EGFR/Rluc8 + Grb2/Venus + pcDNA3	7.57±0.13 (n = 6)	ND (n = 4)
EGFR/Rluc8 + Grb2/Venus + HER3	7.73±0.15 (n = 5)	7.41±0.25 (n = 5)
HER3/Rluc8 + Grb2/Venus + pcDNA3	ND (n = 4)	ND (n = 5)
HER3/Rluc8 + Grb2/Venus + EGFR	7.55±0.15 (n = 9)	7.46±0.14 (n = 5)

Values represent mean ± SEM of multiple independent experiments as indicated (n).

### Effect of EGFR inhibition on EGF- and HRG-induced receptor-Grb2 interaction

**Figure 4:**
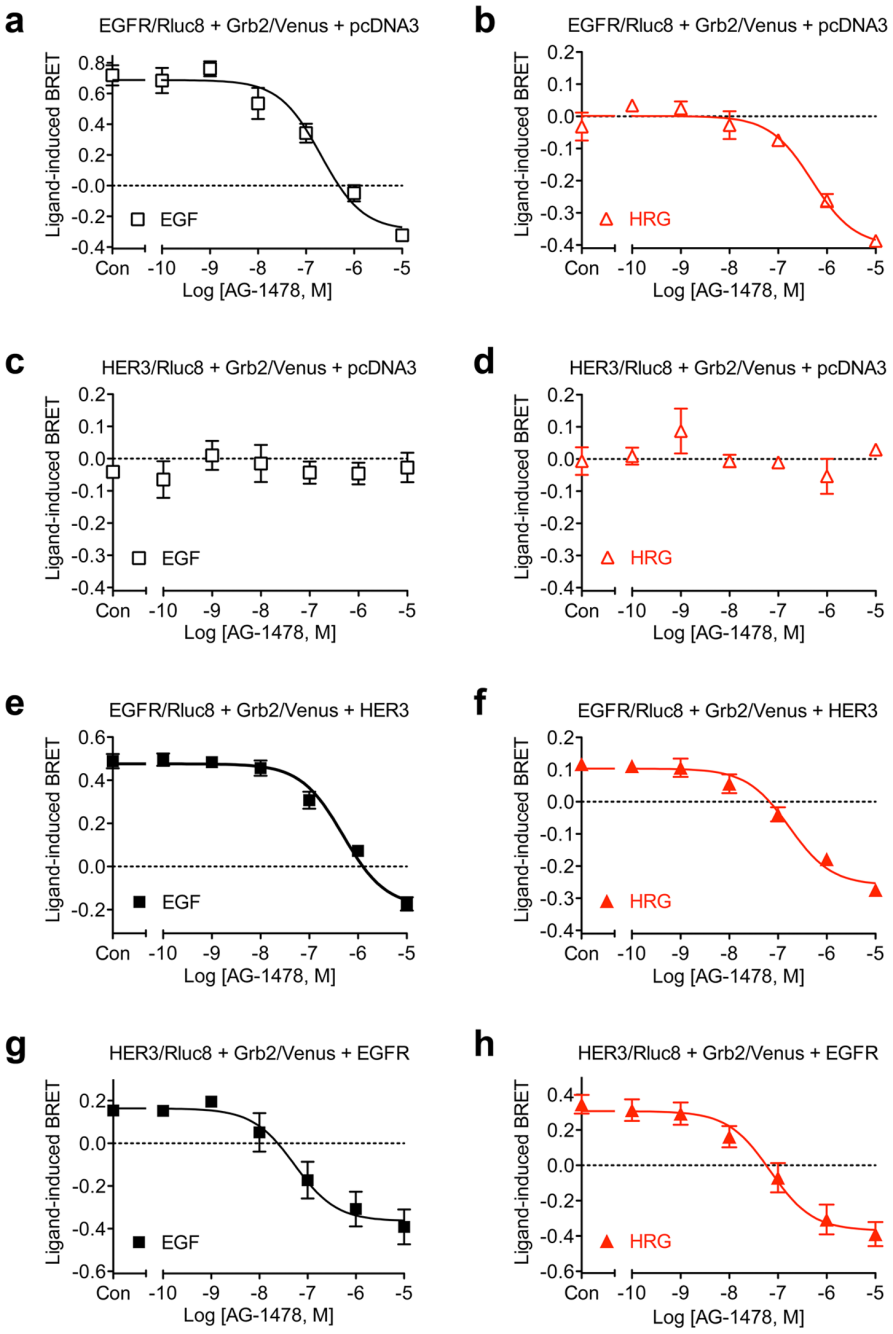
Dose-response analysis of AG-1478-mediated inhibition of Grb2 recruitment to complexes containing EGFR and/or HER3. Live HEK293FT cells were treated with 20 nM agonist followed by increasing concentrations of AG-1478 or vehicle control (Con) after about 20 minutes. The data shown were generated about 60 minutes following initial agonist stimulation. Cells expressing EGFR/Rluc8 and Grb2/Venus treated with EGF (**a**) or HRG (**b**), cells expressing HER3/Rluc8 and Grb2/Venus treated with EGF (**c**) or HRG (**d**), cells expressing EGFR/Rluc8, Grb2/Venus and HER3 treated with EGF (**e**) or HRG (**f**), and cells expressing HER3/Rluc8, Grb2/Venus and EGFR treated with EGF (**g**) or HRG (**h**). Data represent mean ± SEM of 3–5 independent experiments.

To confirm the link between the ligand-induced Grb2 recruitment and receptor activation we examined the effect of EGFR inhibitor AG-1478, known to inhibit EGFR activation, phosphorylation and downstream signaling [37]. We studied the effect of increasing doses of AG-1478 on the recruitment of Grb2 to EGFR and HER3 homo- and heteromers. As shown in Figure 4a, increasing concentrations of AG-1478 completely inhibited EGF-induced BRET measured in cells co-expressing EGFR/Rluc8 and Grb2/Venus. Surprisingly, we observed that at 10 µM of AG-1478, the inhibitory effect was such that the BRET signal was brought down below baseline, consistent with inhibition of constitutive BRET between EGFR/Rluc8 and Grb2/Venus (Figure 4a). This observation is supported by the data shown in Figure 4b, where AG-1478 dramatically reduced the BRET signal below the basal level in a dose-dependent manner with cells co-expressing EGFR/Rluc8 and Grb2/Venus and pretreated with HRG (which as expected and shown previously, did not promote a BRET increase in these cells). Moreover, when the curves in Figure 4a and b are compared, the pIC_50_ values for AG-1478 on both constitutive and EGF-induced BRET are similar (Table 2). Together these data indicate that AG-1478 inhibits both constitutive as well as EGF-induced EGFR-Grb2 interaction. The effect on the constitutive interaction suggests that AG-1478 acts as an inverse agonist on EGFR homomers with respect to their interaction with Grb2. The inhibitory effect of AG-1478 on the basal BRET signal cannot be explained by a non-specific effect on the BRET signal since the increasing concentrations of AG-1478 had no effect on BRET measured in cells co-expressing HER3/Rluc8 and Grb2/Venus and pre-treated or not with 20 nM of either EGF (Figure 4c) or HRG (Figure 4d). These observations also confirm that HER3 homomers are not interacting with Grb2 in the absence of EGFR co-expression.

**Table 2:** pIC_50_ values for AG-1478 determined in the presence of 20 nM of agonist.

*Transfections*	*EGF stimulation*	*HRG stimulation*
EGFR/Rluc8 + Grb2/Venus + pcDNA3	6.68±0.17 (n = 3)	6.31±0.08 (n = 3)
HER3/Rluc8 + Grb2/Venus + pcDNA3	ND (n = 3)	ND (n = 3)
EGFR/Rluc8 + Grb2/Venus + HER3	6.35±0.11 (n = 4)	6.76±0.11 (n = 5)
HER3/Rluc8 + Grb2/Venus + EGFR	7.26±0.27 (n = 5)	7.13±0.24 (n = 5)

Values represent mean ± SEM of multiple independent experiments as indicated (n).

In EGFR-HER3 heteromer configurations, increasing doses of AG-1478 inhibited BRET signals in both EGF- (Figure 4e and g) and HRG-treated (Figure 4f and h) cells. The pIC_50_ values for AG-1478 inhibiting EGFR/Rluc8-Grb2/Venus proximity (induced and constitutive) was not significantly affected by the co-expression of HER3 (Table 2). This may well be because much of the inhibition of the BRET signal with the EGFR/Rluc8, Grb2/Venus and HER3 combination is actually inhibition of EGFR/Rluc8 homomers interacting with Grb2/Venus. In contrast, EGFR co-expression was required in order for AG-1478 inhibition of BRET between HER3/Rluc8 and Grb2/Venus to be observed (Figure 4g and h), with pIC_50_ values not significantly different from those observed between EGFR/Rluc8 homomers and Grb2/Venus (Table 2).

**Figure 5:**
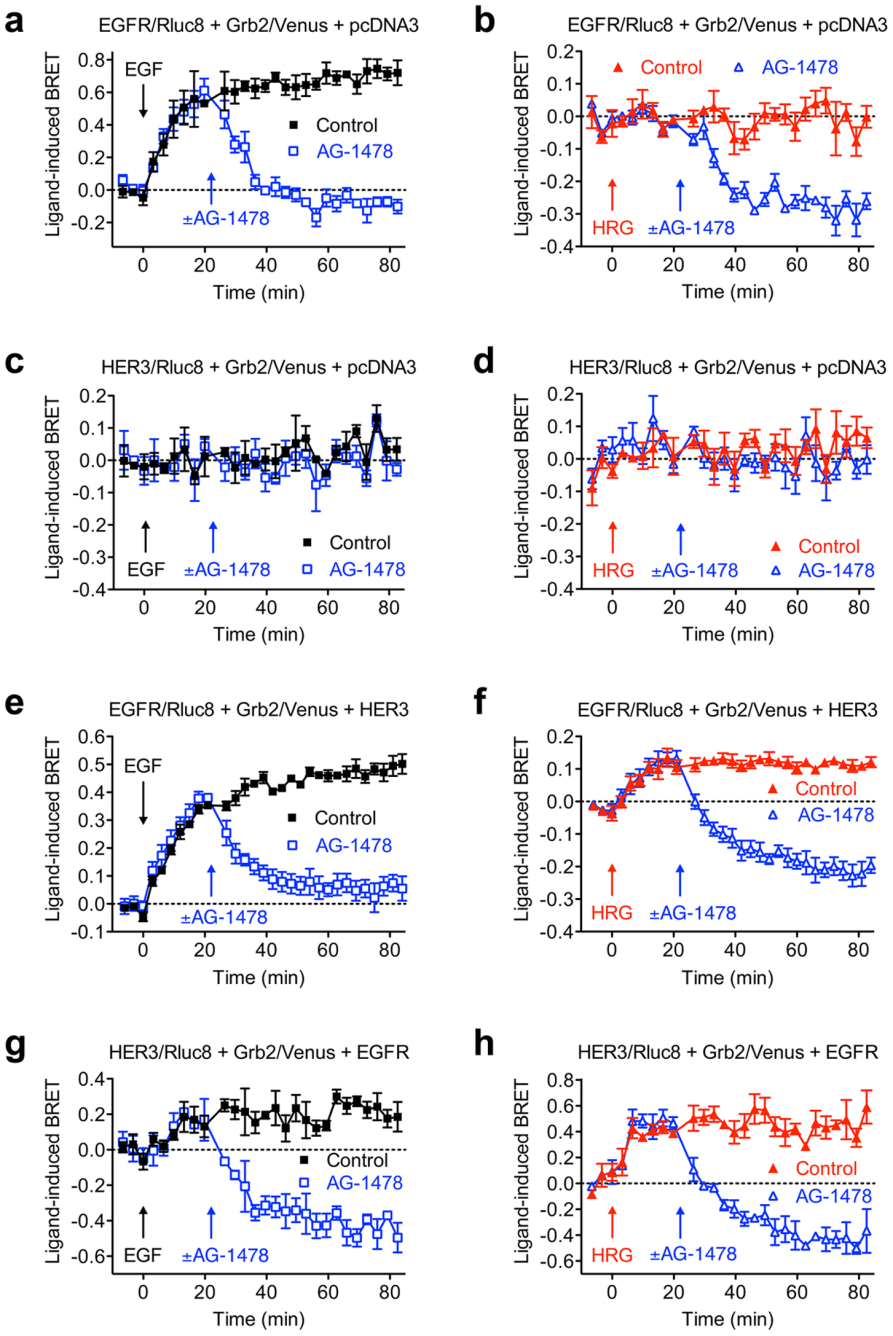
Kinetic analysis of AG-1478-mediated inhibition of Grb2 recruitment to complexes containing EGFR and/or HER3. HEK293FT cells were treated with 20 nM agonist followed by 1 µM AG-1478 or vehicle (control) after about 20 minutes as indicated. Cells expressing EGFR/Rluc8 and Grb2/Venus treated with EGF (**a**) or HRG (**b**), cells expressing HER3/Rluc8 and Grb2/Venus treated with EGF (**c**) or HRG (**d**), cells expressing EGFR/Rluc8, Grb2/Venus and HER3 treated with EGF (**e**) or HRG (**f**), and cells expressing HER3/Rluc8, Grb2/Venus and EGFR treated with EGF (**g**) or HRG (**h**). BRET was measured in real-time and live cells before and after stimulation with the agonists, and before and after stimulation with AG-1478 as indicated. Data represent mean ± SEM of 3 independent experiments.

Kinetic profiles of the inhibitory effect from 1 µM of AG-1478 are shown in Figure 5. Cells were prestimulated with 20 nM of either EGF or HRG and therefore AG-1478 was seen to inhibit both the EGF-induced (Figure 5a) and constitutive (Figure 5b) interaction of EGFR homomers with Grb2, in a time-dependent manner. Indeed, this submaximal dose of AG-1478 (see Figure 4) illustrates that, even though the pIC_50_ values are similar (Table 2), receptor activation by EGF increases the dose of AG-1478 required to substantially reduce the constitutive BRET signal (Figure 4a compared to 4b). Therefore, this means that 1 µM of AG-1478 is able to effectively block the EGF-induced BRET, but is not enough to affect the constitutive BRET (Figure 5a), in contrast to the effect of this dose in the absence of EGF-stimulation (Figure 5b). This is due to there being a difference between the baseline ligand-induced BRET signal and the true baseline at which there is no constitutive or ligand-induced receptor activation. Consequently, the baseline ligand-induced BRET ratio changes relative to the dose-response curve's minimum and maximum depending upon how much ligand-induced receptor activation occurs. As expected, AG-1478 had no effect on HER3-Grb2 interaction (Figure 5c and d).

In cells co-expressing EGFR/Rluc8, Grb2/Venus and HER3, 1 µM of AG-1478 abolished EGF- (Figure 5e) and HRG- (Figure 5f) induced BRET and largely decreased the constitutive BRET signal between EGFR/Rluc8 and Grb2/Venus in cells stimulated with HRG, but not EGF (Figure 5f). This difference is again due to the increased receptor activation observed with EGF compared with HRG, in addition to constitutive receptor activation, and the effect this has on the dose-response curves (Figure 4e compared to 4f), in a similar manner to that discussed above. In cells co-expressing HER3/Rluc8, Grb2/Venus and EGFR, 1 µM of AG-1478 completely blocked EGF- (Figure 5g) and HRG- (Figure 5h) induced BRET and strongly decreased the constitutive BRET signal between HER3/Rluc8 and Grb2/Venus within the EGFR-HER3 heteromers. All together, these kinetics clearly confirm the link between the activation of EGFR homomers, as well as EGFR-HER3 heteromers, and their interaction with Grb2.

### Importance of the Grb2 binding sites in HER3 for Grb2 interaction with EGFR-HER3 heteromers

**Figure 6:**
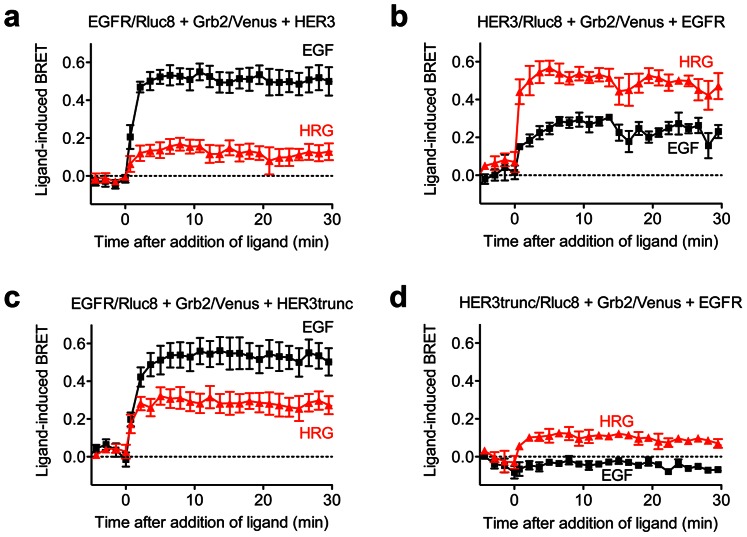
Kinetic analysis of Grb2 recruitment to complexes containing EGFR and full length or truncated HER3. HEK293FT cells were treated with 1 µM EGF or HRG. Cells expressing EGFR/Rluc8, Grb2/Venus and HER3 (**a**), cells expressing HER3/Rluc8, Grb2/Venus and EGFR (**b**), cells expressing EGFR/Rluc8, Grb2/Venus and truncated HER3 (HER3trunc; **c**), and cells expressing HER3trunc/Rluc8, Grb2/Venus and EGFR (**d**). BRET was measured in real-time and live cells before and after stimulation with the agonists as indicated. Data represent mean ± SEM of 4 independent experiments.

Activation of the EGFR-HER3 heteromer could result in recruitment of Grb2 to interact with either the EGFR or HER3 protomer, or both. To investigate this we generated a truncated mutant of HER3 lacking its C-terminal region, and therefore all of the putative binding sites for Grb2, and compared this with the full length receptor using RTK-HIT. The positive controls confirmed that EGF and HRG induced BRET signals between EGFR/Rluc8 (Figure 6a) or HER3/Rluc8 (Figure 6b) and Grb2/Venus within the EGFR-HER3 heteromer. The co-expression of the truncated rather than full length HER3 was not detrimental to the EGF- or HRG-induced BRET signal between EGFR/Rluc8 and Grb2/Venus (Figure 6c). Indeed, if anything the effect of HRG, but not EGF, appears to be even stronger with truncated HER3 (Figure 6c) compared to full length HER3 (Figure 6a). The lack of effect with EGF treatment may be because this signal is predominantly from EGFR/Rluc8 homomers recruiting Grb2/Venus. In contrast, the truncation of HER3 completely abolished the EGF-induced BRET signal between HER3/Rluc8 and Grb2/Venus even in the presence of EGFR (Figure 6d), whilst a small but discernible HRG-induced BRET signal was still observed.

## Discussion

In this study we describe a proximity-based assay, RTK-HIT, to investigate the heteromerization between EGFR and HER3 in real-time and live HEK293FT cells. RTK-HIT is an adaptation of GPCR-HIT previously described to identify and profile GPCR heteromers [28], [29], [31]. By using RTK-HIT we demonstrate the existence of functional interaction between EGFR and HER3 in live HEK293FT cells. The heteromerization between the different members of the HER family has been known for many years, but their link with cancer means that the study of these receptor complexes continues to be of considerable importance, including development of novel approaches to investigate their pharmacological and signaling characteristics. Indeed, while EGFR is the prototypical RTK, with its ability to bind EGF and activate multiple downstream signaling pathways via the phosphorylation of various specific tyrosine residues in its C-terminal domain, the situation with HER2 and HER3 is completely different and intriguing. HER2 is considered to be an orphan/silent receptor with no specific ligand known, yet it seems to exhibit normal kinase activity when heteromerizing with other HER members. In contrast, HER3 is known to be the specific receptor of HRG, but appears to have lost its tyrosine kinase activity suggesting that its heteromerization with other family members is a prerequisite for activation of specific signaling pathways [4], [8], [10], [17], [19]–[21], [23]. Thus, better understanding of the heteromerization between HER3 and other members of the HER family is an important research aim.

Our study shows that RTK-HIT is not only a method to detect the proximity between different receptors (implying physical interaction) but more importantly, it allows the investigation of the functional consequences and features of such receptor complexes (functional interaction) and their pharmacological profiling. Indeed, we have clearly demonstrated that the interaction of HER3 with Grb2 requires the heteromerization of HER3 with another receptor, such as EGFR. Furthermore, the utilization of different BRET configurations of untagged or Rluc8-tagged (BRET donor) EGFR or HER3 co-expressed with Grb2/Venus (BRET acceptor), led us to demonstrate that the stimulation of either EGFR or HER3 within the EGFR-HER3 heteromer was sufficient to promote Grb2 recruitment. These observations appear to contradict the canonical model of EGFR family activation that involves tethering of the dimerization arm of domain II in the unliganded receptor and upon ligand binding, this arm is released to enable interaction with the partner receptor and consequent activation of the signaling complex [38]. This was thought to necessitate ligand binding to both protomers of the dimer pair in order for both dimerization arms to be released for interaction. However, the recent work of Liu et al. has provided evidence that a single ligand is sufficient to activate EGFR dimers [39], suggesting that interaction with a liganded receptor can result in untethering and extension of an unliganded receptor such that it can participate in a signaling complex. Notably, we and others have provided evidence for allosteric activation of unliganded receptors for GPCRs (see [29] for example), and therefore there is also precedence from this other major class of membrane receptors for such transactivation to occur.

The selective inhibition of EGFR using AG-1478 completely abolished both the constitutive as well as EGF/HRG-induced Grb2 interaction with the heteromer. Therefore, our data with AG-1478 clearly demonstrate that (i) EGFR homomers are constitutively interacting with Grb2 in HEK293FT cells, which may be due to some constitutive activity of the receptor, at least with regard to the Grb2 pathway in this cell line, (ii) EGF- as well as HRG-induced Grb2 recruitment strictly depends on receptor activation (constitutive or ligand-induced), and (iii) the recruitment of Grb2 to HER3 is observed only when HER3 is engaged in a heteromer complex with another receptor subtype such as EGFR. Furthermore, our data with AG-1478, which only inhibits EGFR activity, suggest that the activation of the EGFR-HER3 heteromer occurs via a transactivation mechanism whereby the EGFR protomer constitutes the key element for HER3 to interact with Grb2 and activate its dependent signaling pathway.

Our findings with the truncated HER3 indicate that Grb2 is able to bind to the EGFR protomer of the EGFR-HER3 heteromer even following HRG activation of HER3, implying transactivation across the heteromer complex. However, even if HER3 transactivates EGFR, it is still surprising that removal of the putative Grb2 binding sites from the C-terminus of HER3 did not prevent Grb2 recruitment to the EGFR-HER3 heteromer. This is because the kinase activity of the activated EGFR protomer would be expected to phosphorylate the HER3 C-terminus. The notion that the activated EGFR protomer could phosphorylate its own C-terminus goes against all current models of EGFR activation. One possibility is that heteromerization somehow re-establishes the kinase activity of HER3, or the combination of EGFR-HER3 enables the kinase activity of EGFR to be utilized, so that an activated protomer could phosphorylate the C-terminus of its unliganded partner protomer. HRG-induced activation of truncated HER3 could then result in phosphorylation of the EGFR C-terminus and consequent recruitment of Grb2/Venus, resulting in a stronger BRET signal when the EGFR is Rluc8-tagged, but still enabling a signal to be seen when the truncated HER3 is Rluc8-tagged and energy is transferred across the complex. This is at least consistent with the data seen in Figure 6c and 6d respectively.

An alternative and perhaps more likely explanation could be provided by the formation of higher order complexes. For example, our data would be consistent with a single ligand activating one protomer in an EGFR-HER3 heterodimer (perhaps preformed), the result being transactivation and release of the dimerization arm from the unliganded protomer. This unliganded protomer could in turn interact with and phosphorylate a third protomer, potentially in another activated heterodimer. Two heterodimers interacting to form a tetramer is conceptually very similar to the canonical model of monomers interacting to form homodimers, but with the (preformed) heterodimers effectively acting like monomer units. The notion of preformed RTK heterodimers undergoing a conformational change upon binding ligand is not new [12], [13]. Furthermore, EGFR activation resulting in a dimer to tetramer transition has been proposed by Clayton et al. [15], [16] and Zhang et al. have also recently presented a model of HER2-HER3 complex formation as a tetramer of side-by-side heterodimers [10]. Therefore, this model of EGFR-HER3 activation in many ways reconciles the canonical activation model with the concept of preformed heterodimers and active tetramer/higher order complexes.

It is not inconceivable that, when the HER3 is not truncated, there is competition between phosphorylation of the HER3 C-terminus and the second EGFR protomer C-terminus. This may explain the increase in HRG-induced BRET signal observed in Figure 6c compared to 6a, keeping in mind that Grb2/Venus recruitment directly to the EGFR/Rluc8 will result in a greater BRET signal than recruitment to untagged HER3 proximal to EGFR/Rluc8 (due to energy transfer dramatically increasing with increased proximity).

With the opposite BRET orientation, HER3 truncation means that Grb2/Venus can only bind to EGFR in the HER3trunc/Rluc8-EGFR complex, which results in less donor-acceptor proximity than binding to HER3/Rluc8. Again, a higher order complex could enable recruitment of Grb2/Venus to a second untagged EGFR protomer rather than HER3/Rluc8, which would be expected to result in a lower BRET signal as is indeed observed in Figure 6d compared to 6b.

These data also demonstrate the importance of the HER3 C-terminal region for EGF-induced Grb2 signaling by the EGFR-HER3 heteromer, as shown for PI3-kinase/Akt signaling mediated by HER2-HER3 [40], as HER3 truncation totally abolished the EGF-induced BRET signal. It is interesting however, that the HER3 C-terminus is not required for HRG-induced Grb2 recruitment to the EGFR-HER3 heteromer. Together, our data are consistent with the allosteric transactivation mechanism involved in the activation of the EGFR-HER3 heteromer as previously proposed for the HER family [41]. The importance of the kinase domain of HER3 (not removed by the truncation) for EGFR-dependent Grb2 interaction is in agreement with the recent structural study showing that although the HER3 kinase domain is not functional in terms of kinase activity, it can activate the EGFR kinase domain by formation of the asymmetric dimer [25]. In that study, the authors proposed that the kinase domain of HER3 is always in the favorable form to engage and activate the kinase domains of the other members of the family [25].

From the signaling point of view, our findings are consistent with previous studies reporting that cells co-expressing EGFR and HER3 show an EGF-dependent HER3 phosphorylation [42], [43]. Also, HER3 has been reported to promote EGF-dependent PI3-kinase activation in some cell lines co-expressing both EGFR and HER3 [43]. Moreover, many studies reported that HER3 is principally coupled to the PI3-kinase/Akt pathway through its binding with the p85 subunit of PI3-kinase and this was essentially mediated by the HER2-HER3 heteromer [23], [44]–[46]. Similarly, HRG-stimulated interaction of Shc with HER3 has been shown to be mediated by the HER2-HER3 complex [47]. Here we describe a mechanism by which HER3 interacts with the adaptor protein Grb2 via its heteromerization with EGFR, linking HER3 with the Ras/mitogen-activated protein kinase pathway. This demonstrates the importance of heteromerization for HER3-mediated signaling and further illustrates the diversity of HER-dependent signaling being mediated by heteromerization. In addition, the importance of EGFR for HRG-induced HER3-Grb2 interaction indicates that EGFR can also be considered as an allosteric activator of other members of the HER family through heteromerization.

In addition, our data provide evidence for the importance of EGFR-HER3 heteromerization in physiology and pathology since the functional interaction between EGFR and HER3 and their possible crosstalk at the level of downstream signaling is not clear yet. Indeed, many studies on the HER family have pointed out the central role of HER2 in regulating and diversifying EGFR and HER3 function and their balance via different patterns of heteromerization between these three members [17], [20], [21], [48]–[50]. For instance, a recent study on human mammary epithelial cells has reported that EGFR activation can induce HER3 phosphorylation and activation only in the presence of HER2 but the stimulation of the HER3 pathway by its ligand did not induce EGFR activation regardless of the presence of HER2 [51]. Moreover, EGF and HRG have been shown to induce differential signaling pathways by affecting the dynamic of receptor homomers versus heteromers between EGFR, HER2 and HER3 where HER2 seems to be the determining element within the system [50]. However, our results support all the recent studies showing a role of HER3 in the regulation of HER function and signaling through its heteromerization with other members including EGFR [11], [25]–[27]. The importance of the EGFR-HER3 heteromerization we have described here has been demonstrated by the recent study showing a beneficial effect of a dual-action antibody targeting both EGFR and HER3 [26], [27]. Moreover, the inhibition of HER3 has been shown to be more relevant than the inhibition of EGFR and HER2 in breast [11] and lung [52] cancer models.

As with GPCR-HIT, the RTK Heteromer Investigation Technology (RTK-HIT) utilized in this study can be performed using a range of proximity-based assays including bioluminescence resonance energy transfer (BRET), fluorescence resonance energy transfer (FRET), bimolecular fluorescence complementation (BiFC), bimolecular luminescence complementation (BiLC), enzyme fragment complementation (EFC), and the protease-cleaved transcription factor assay system known as Tango™ [30], [36]. Advantages and disadvantages of these different reporter systems have been discussed previously [36]. Finally, the ligand-dependent nature of RTK-HIT, whichever assay platform is utilized, is an asset for its application in pharmacological studies as well as drug discovery programs. Indeed, the development of new cell-based assays to assess the heteromerization of the HER family, and profile their pharmacology and signaling in real-time and live cells, is of great interest since many studies have shown that inhibition of HER family-mediated signaling has considerable potential for cancer therapeutics [26], [27], [53].

## References

[1] Kamath S, Buolamwini JK (2006) Targeting EGFR and HER-2 receptor tyrosine kinases for cancer drug discovery and development. Med Res Rev 26: 569–594. 16788977 10.1002/med.20070

[2] Yarden Y, Sliwkowski MX (2001) Untangling the ErbB signalling network. Nat Rev Mol Cell Biol 2: 127–137. 11252954 10.1038/35052073

[3] Hynes NE, MacDonald G (2009) ErbB receptors and signaling pathways in cancer. Curr Opin Cell Biol 21: 177–184. 19208461 10.1016/j.ceb.2008.12.010

[4] Linggi B, Carpenter G (2006) ErbB receptors: new insights on mechanisms and biology. Trends Cell Biol 16: 649–656. 17085050 10.1016/j.tcb.2006.10.008

[5] Olayioye MA, Neve RM, Lane HA, Hynes NE (2000) The ErbB signaling network: receptor heterodimerization in development and cancer. EMBO J 19: 3159–3167. 10880430 10.1093/emboj/19.13.3159 PMC313958

[6] Way TD, Lin JK (2005) Role of HER2/HER3 co-receptor in breast carcinogenesis. Future Oncol 1: 841–849. 16556064 10.2217/14796694.1.6.841

[7] Freeman MR (2004) HER2/HER3 heterodimers in prostate cancer: Whither HER1/EGFR? Cancer Cell 6: 427–428. 15542423 10.1016/j.ccr.2004.10.018

[8] Wallasch C, Weiss FU, Niederfellner G, Jallal B, Issing W, et al (1995) Heregulin-dependent regulation of HER2/neu oncogenic signaling by heterodimerization with HER3. EMBO J 14: 4267–4275. 7556068 10.1002/j.1460-2075.1995.tb00101.x PMC394510

[9] Klapper LN, Kirschbaum MH, Sela M, Yarden Y (2000) Biochemical and clinical implications of the ErbB/HER signaling network of growth factor receptors. Adv Cancer Res 77: 25–79. 10549355

[10] Zhang Q, Park E, Kani K, Landgraf R (2012) Functional isolation of activated and unilaterally phosphorylated heterodimers of ERBB2 and ERBB3 as scaffolds in ligand-dependent signaling. Proc Natl Acad Sci U S A 109: 13237–13242. 22733765 10.1073/pnas.1200105109 PMC3421218

[11] Lee-Hoeflich ST, Crocker L, Yao E, Pham T, Munroe X, et al (2008) A central role for HER3 in HER2-amplified breast cancer: implications for targeted therapy. Cancer Res 68: 5878–5887. 18632642 10.1158/0008-5472.CAN-08-0380

[12] Liu P, Sudhaharan T, Koh RM, Hwang LC, Ahmed S, et al (2007) Investigation of the dimerization of proteins from the epidermal growth factor receptor family by single wavelength fluorescence cross-correlation spectroscopy. Biophys J 93: 684–698. 17468161 10.1529/biophysj.106.102087 PMC1896234

[13] Tao RH, Maruyama IN (2008) All EGF(ErbB) receptors have preformed homo- and heterodimeric structures in living cells. J Cell Sci 121: 3207–3217. 18782861 10.1242/jcs.033399

[14] Bjorkelund H, Gedda L, Barta P, Malmqvist M, Andersson K (2011) Gefitinib induces epidermal growth factor receptor dimers which alters the interaction characteristics with (1)(2)(5)I-EGF. PLoS One 6: e24739. 21931838 10.1371/journal.pone.0024739 PMC3171474

[15] Clayton AH, Orchard SG, Nice EC, Posner RG, Burgess AW (2008) Predominance of activated EGFR higher-order oligomers on the cell surface. Growth Factors 26: 316–324. 18937111 10.1080/08977190802442187

[16] Clayton AH, Walker F, Orchard SG, Henderson C, Fuchs D, et al (2005) Ligand-induced dimer-tetramer transition during the activation of the cell surface epidermal growth factor receptor-A multidimensional microscopy analysis. J Biol Chem 280: 30392–30399. 15994331 10.1074/jbc.M504770200

[17] Pinkas-Kramarski R, Soussan L, Waterman H, Levkowitz G, Alroy I, et al (1996) Diversification of Neu differentiation factor and epidermal growth factor signaling by combinatorial receptor interactions. EMBO J 15: 2452–2467. 8665853 PMC450177

[18] Tzahar E, Pinkas-Kramarski R, Moyer JD, Klapper LN, Alroy I, et al (1997) Bivalence of EGF-like ligands drives the ErbB signaling network. EMBO J 16: 4938–4950. 9305636 10.1093/emboj/16.16.4938 PMC1170129

[19] Zhang K, Sun J, Liu N, Wen D, Chang D, et al (1996) Transformation of NIH 3T3 cells by HER3 or HER4 receptors requires the presence of HER1 or HER2. J Biol Chem 271: 3884–3890. 8632008

[20] Banappagari S, Corti M, Pincus S, Satyanarayanajois S (2012) Inhibition of protein-protein interaction of HER2-EGFR and HER2-HER3 by a rationally designed peptidomimetic. J Biomol Struct Dyn 30: 594–606. 22731912 10.1080/07391102.2012.687525 PMC3572747

[21] Earp HS, Dawson TL, Li X, Yu H (1995) Heterodimerization and functional interaction between EGF receptor family members: a new signaling paradigm with implications for breast cancer research. Breast Cancer Res Treat 35: 115–132. 7612898 10.1007/BF00694752

[22] Berger MB, Mendrola JM, Lemmon MA (2004) ErbB3/HER3 does not homodimerize upon neuregulin binding at the cell surface. FEBS Lett 569: 332–336. 15225657 10.1016/j.febslet.2004.06.014

[23] Campbell MR, Amin D, Moasser MM (2010) HER3 comes of age: new insights into its functions and role in signaling, tumor biology, and cancer therapy. Clin Cancer Res 16: 1373–1383. 20179223 10.1158/1078-0432.CCR-09-1218 PMC2831167

[24] Sierke SL, Cheng K, Kim HH, Koland JG (1997) Biochemical characterization of the protein tyrosine kinase homology domain of the ErbB3 (HER3) receptor protein. Biochem J 322 (Pt 3): 757–763. 10.1042/bj3220757 PMC1218252 9148746

[25] Jura N, Shan Y, Cao X, Shaw DE, Kuriyan J (2009) Structural analysis of the catalytically inactive kinase domain of the human EGF receptor 3. Proc Natl Acad Sci U S A 106: 21608–21613. 20007378 10.1073/pnas.0912101106 PMC2791034

[26] Kamath AV, Lu D, Gupta P, Jin D, Xiang H, et al (2012) Preclinical pharmacokinetics of MEHD7945A, a novel EGFR/HER3 dual-action antibody, and prediction of its human pharmacokinetics and efficacious clinical dose. Cancer Chemother Pharmacol 69: 1063–1069. 22203367 10.1007/s00280-011-1806-6

[27] Schaefer G, Haber L, Crocker LM, Shia S, Shao L, et al (2011) A two-in-one antibody against HER3 and EGFR has superior inhibitory activity compared with monospecific antibodies. Cancer Cell 20: 472–486. 22014573 10.1016/j.ccr.2011.09.003

[28] Ayoub MA, Pfleger KD (2010) Recent advances in bioluminescence resonance energy transfer technologies to study GPCR heteromerization. Curr Opin Pharmacol 10: 44–52. 19897419 10.1016/j.coph.2009.09.012

[29] Mustafa S, See HB, Seeber RM, Armstrong SP, White CW, et al (2012) Identification and profiling of novel alpha1A-adrenoceptor-CXC chemokine receptor 2 heteromer. J Biol Chem 287: 12952–12965. 22371491 10.1074/jbc.M111.322834 PMC3340001

[30] Mustafa S, Pfleger KD (2011) G protein-coupled receptor heteromer identification technology: identification and profiling of GPCR heteromers. J Lab Autom 16: 285–291. 21764024 10.1016/j.jala.2011.03.002

[31] See HB, Seeber RM, Kocan M, Eidne KA, Pfleger KD (2011) Application of G protein-coupled receptor-heteromer identification technology to monitor beta-arrestin recruitment to G protein-coupled receptor heteromers. Assay Drug Dev Technol 9: 21–30. 21133678 10.1089/adt.2010.0336 PMC3034639

[32] Porrello ER, Pfleger KD, Seeber RM, Qian H, Oro C, et al (2011) Heteromerization of angiotensin receptors changes trafficking and arrestin recruitment profiles. Cell Signal 23: 1767–1776. 21740964 10.1016/j.cellsig.2011.06.011

[33] Watts AO, van Lipzig MM, Jaeger WC, Seeber RM, van Zwam M, et al (2013) Identification and profiling of CXCR3-CXCR4 chemokine receptor heteromer complexes. Br J Pharmacol 168: 1662–1674. 23170857 10.1111/bph.12064 PMC3605874

[34] Pfleger KD, Dromey JR, Dalrymple MB, Lim EM, Thomas WG, et al (2006) Extended bioluminescence resonance energy transfer (eBRET) for monitoring prolonged protein-protein interactions in live cells. Cell Signal 18: 1664–1670. 16492395 10.1016/j.cellsig.2006.01.004

[35] Pfleger KD, Eidne KA (2006) Illuminating insights into protein-protein interactions using bioluminescence resonance energy transfer (BRET). Nat Methods 3: 165–174. 16489332 10.1038/nmeth841

[36] Johnstone EK, Pfleger KD (2012) Receptor-Heteromer Investigation Technology and its application using BRET. Front Endocrinol (Lausanne) 3: 101. 22936924 10.3389/fendo.2012.00101 PMC3424490

[37] Osherov N, Levitzki A (1994) Epidermal-growth-factor-dependent activation of the src-family kinases. Eur J Biochem 225: 1047–1053. 7525285 10.1111/j.1432-1033.1994.1047b.x

[38] Ferguson KM, Berger MB, Mendrola JM, Cho HS, Leahy DJ, et al (2003) EGF activates its receptor by removing interactions that autoinhibit ectodomain dimerization. Mol Cell 11: 507–517. 12620237 10.1016/s1097-2765(03)00047-9

[39] Liu P, Cleveland TE IV, Bouyain S, Byrne PO, Longo PA, et al (2012) A single ligand is sufficient to activate EGFR dimers. Proc Natl Acad Sci U S A 109: 10861–10866. 22699492 10.1073/pnas.1201114109 PMC3390854

[40] Choi BK, Cai X, Yuan B, Huang Z, Fan X, et al (2012) HER3 intracellular domains play a crucial role in HER3/HER2 dimerization and activation of downstream signaling pathways. Protein Cell 3: 781–789. 22983903 10.1007/s13238-012-2065-y PMC4875347

[41] Zhang X, Gureasko J, Shen K, Cole PA, Kuriyan J (2006) An allosteric mechanism for activation of the kinase domain of epidermal growth factor receptor. Cell 125: 1137–1149. 16777603 10.1016/j.cell.2006.05.013

[42] Kim HH, Sierke SL, Koland JG (1994) Epidermal growth factor-dependent association of phosphatidylinositol 3-kinase with the erbB3 gene product. J Biol Chem 269: 24747–24755. 7929151

[43] Soltoff SP, Carraway KL 3rd, Prigent SA, Gullick WG, Cantley LC (1994) ErbB3 is involved in activation of phosphatidylinositol 3-kinase by epidermal growth factor. Mol Cell Biol 14: 3550–3558. 7515147 10.1128/mcb.14.6.3550-3558.1994 PMC358722

[44] Suenaga A, Takada N, Hatakeyama M, Ichikawa M, Yu X, et al (2005) Novel mechanism of interaction of p85 subunit of phosphatidylinositol 3-kinase and ErbB3 receptor-derived phosphotyrosyl peptides. J Biol Chem 280: 1321–1326. 15520002 10.1074/jbc.M410436200

[45] Balz LM, Bartkowiak K, Andreas A, Pantel K, Niggemann B, et al (2012) The interplay of HER2/HER3/PI3K and EGFR/HER2/PLC-gamma1 signalling in breast cancer cell migration and dissemination. J Pathol 227: 234–244. 22262199 10.1002/path.3991

[46] Carraway KL 3rd, Soltoff SP, Diamonti AJ, Cantley LC (1995) Heregulin stimulates mitogenesis and phosphatidylinositol 3-kinase in mouse fibroblasts transfected with erbB2/neu and erbB3. J Biol Chem 270: 7111–7116. 7535767 10.1074/jbc.270.13.7111

[47] Vijapurkar U, Cheng K, Koland JG (1998) Mutation of a Shc binding site tyrosine residue in ErbB3/HER3 blocks heregulin-dependent activation of mitogen-activated protein kinase. J Biol Chem 273: 20996–21002. 9694850 10.1074/jbc.273.33.20996

[48] Shankaran H, Wiley HS, Resat H (2006) Modeling the effects of HER/ErbB1-3 coexpression on receptor dimerization and biological response. Biophys J 90: 3993–4009. 16533841 10.1529/biophysj.105.080580 PMC1459488

[49] Tzahar E, Waterman H, Chen X, Levkowitz G, Karunagaran D, et al (1996) A hierarchical network of interreceptor interactions determines signal transduction by Neu differentiation factor/neuregulin and epidermal growth factor. Mol Cell Biol 16: 5276–5287. 8816440 10.1128/mcb.16.10.5276 PMC231527

[50] Gamett DC, Pearson G, Cerione RA, Friedberg I (1997) Secondary dimerization between members of the epidermal growth factor receptor family. J Biol Chem 272: 12052–12056. 9115272 10.1074/jbc.272.18.12052

[51] Zhang Y, Opresko L, Shankaran H, Chrisler WB, Wiley HS, et al (2009) HER/ErbB receptor interactions and signaling patterns in human mammary epithelial cells. BMC Cell Biol 10: 78. 19878579 10.1186/1471-2121-10-78 PMC2776588

[52] Sakai K, Yokote H, Murakami-Murofushi K, Tamura T, Saijo N, et al (2007) Pertuzumab, a novel HER dimerization inhibitor, inhibits the growth of human lung cancer cells mediated by the HER3 signaling pathway. Cancer Sci 98: 1498–1503. 17627612 10.1111/j.1349-7006.2007.00553.x PMC11158426

[53] Britten CD (2004) Targeting ErbB receptor signaling: a pan-ErbB approach to cancer. Mol Cancer Ther 3: 1335–1342. 15486201

